# The physiological functions of iron regulatory proteins in iron homeostasis - an update

**DOI:** 10.3389/fphar.2014.00124

**Published:** 2014-06-13

**Authors:** De-Liang Zhang, Manik C. Ghosh, Tracey A. Rouault

**Affiliations:** Molecular Medicine Program, Eunice Kennedy Shriver National Institute of Child Health and Human Development, National Institute of HealthBethesda, MD, USA

**Keywords:** iron regulatory protein, iron responsive element, erythropoiesis, polycythemia, pulmonary hypertension, iron metabolism

## Abstract

Iron regulatory proteins (IRPs) regulate the expression of genes involved in iron metabolism by binding to RNA stem-loop structures known as iron responsive elements (IREs) in target mRNAs. IRP binding inhibits the translation of mRNAs that contain an IRE in the 5′untranslated region of the transcripts, and increases the stability of mRNAs that contain IREs in the 3′untranslated region of transcripts. By these mechanisms, IRPs increase cellular iron absorption and decrease storage and export of iron to maintain an optimal intracellular iron balance. There are two members of the mammalian IRP protein family, IRP1 and IRP2, and they have redundant functions as evidenced by the embryonic lethality of the mice that completely lack IRP expression (*Irp1*^-/-^/Irp2^-/-^ mice), which contrasts with the fact that *Irp1*^-/-^ and *Irp2*^-/-^ mice are viable. In addition, *Irp2*^-/-^ mice also display neurodegenerative symptoms and microcytic hypochromic anemia, suggesting that IRP2 function predominates in the nervous system and erythropoietic homeostasis. Though the physiological significance of IRP1 had been unclear since *Irp1*^-/-^ animals were first assessed in the early 1990s, recent studies indicate that IRP1 plays an essential function in orchestrating the balance between erythropoiesis and bodily iron homeostasis. Additionally, *Irp1*^-/-^ mice develop pulmonary hypertension, and they experience sudden death when maintained on an iron-deficient diet, indicating that IRP1 has a critical role in the pulmonary and cardiovascular systems. This review summarizes recent progress that has been made in understanding the physiological roles of IRP1 and IRP2, and further discusses the implications for clinical research on patients with idiopathic polycythemia, pulmonary hypertension, and neurodegeneration.

## INTRODUCTION

Iron is an indispensable element for all living organisms. Healthy adults contain 4–5 g of iron, about 65% of which is contained in hemoglobin where it participates in oxygen transport, 30–35% is stored in liver, primarily in the storage protein, ferritin, and 1–2% is found in the form of iron–sulfur clusters or heme in the catalytic centers of numerous essential enzymes and multiprotein complexes such as the mitochondrial respiratory chain complexes, which contain twelve iron–sulfur clusters and seven hemes (Hentze et al., 2004; Darshan et al., 2010; Ganz and Nemeth, 2012b; Rouault, 2013). Due to the essential role of iron *in vivo*, iron deficiency can retard early development and impair cognitive ability of children, and iron deficiency anemia is a common nutrient deficiency disease worldwide. Conversely, because of the chemical reactivity of iron and its ability through Fenton chemistry to generate reactive hydroxyl radicals, which can then oxidize lipids, proteins and DNA, iron overload can damage cells and tissues, and lead to adverse consequences, such as those seen in hemochromatosis and hemolytic anemias. Therefore iron concentration has to be tightly regulated in the tissues and cells of organisms *in vivo*.

Mammals have developed sophisticated mechanisms to maintain appropriate iron concentrations *in vivo.* Thanks to the application of genetic screens and transgenic technology in biomedical research, our understanding of iron homeostasis regulation has advanced significantly in the last 15 years. Iron homeostasis in mammals is mainly regulated by a set of interlocking regulatory systems, including: (i) Hepcidin–ferroportin (FPN1) mediated regulation of serum iron levels, (ii) iron regulatory proteins (IRPs)/iron responsive element (IRE) mediated regulation of intracellular iron homeostasis, (iii) hypoxia inducible factor-2α (HIF2α) mediated transcriptional regulation. These mechanisms regulate iron homeostasis at different levels, and the interaction and cooperation of these mechanisms fine-tunes iron levels *in vivo*. The IRP/IRE machinery post-transcriptionally regulates the expression of target genes according to cellular iron status, providing the fundamental regulation of iron homeostasis at the cellular level. Recently, studies in animal models have also shown that IRPs contribute significantly to systemic iron homeostasis and regulation of erythropoiesis. This review will begin with an overview of mammalian iron homeostasis, and will then focus on the more recent progress made in understanding the roles of IRP1 and IRP2 in cellular and systemic iron homeostasis.

## OVERVIEW OF SYSTEMIC AND CELLULAR IRON METABOLISM

For a healthy adult, about 25–30 mg of iron is needed daily for protein synthesis and cellular regeneration. To meet these requirements, about 90% of iron is acquired from the recycle of senescent red blood cells (RBC) by splenic macrophages, whereas the remaining 10% is absorbed from the diet to compensate for iron loss caused by bleeding, urinary excretion, and sloughing of epithelial and mucous cells (Rigby, 1971). More than 90% of daily iron consumption is used for RBC production in erythropoietic tissues, and the most prominent manifestation of iron deficiency is microcytic anemia (Rigby, 1971). The systemic iron homeostasis is mainly maintained by coordinating iron absorption through the duodenum, iron recycling through splenic macrophages, iron utilization in bone marrow by erythropoiesis, and iron storage in the liver. Because there is no known regulated iron excretion pathway, regulation of intestinal iron absorption plays an important role in maintenance of systemic iron homeostasis.

Mammals can absorb both heme iron and non-heme iron. Iron derived from heme, especially in the people of western societies, is estimated to contribute two thirds of the daily dietary iron absorption (West and Oates, 2008). However, the mechanism for heme absorption is not yet clear. In the last decade, several heme transporters have been identified, including heme carrier protein-1 (HCP1; Qiu et al., 2006; Laftah et al., 2009), HRG-1 (Rajagopal et al., 2008; White et al., 2013), and FLVCR1 and 2 (Quigley et al., 2004; Keel et al., 2008; Duffy et al., 2010), but their significance in intestinal iron absorption remains to be elucidated. For non-heme iron absorption, ferric iron [Fe(III)] in the diet must be reduced by a ferrireductase duodenal cytochrome b561 (Dcytb) to ferrous iron [Fe(II)] before the divalent metal transporter 1 (DMT1, also known as DCT1 or NRAMP2) can transport iron across the apical membrane into the cytosol of duodenal epithelial cells (so-called enterocytes; Gunshin et al., 1997; McKie et al., 2001). Once inside the cells, part of the newly absorbed iron is exported across the basolateral membrane by the iron export protein, ferroportin (FPN1, also known as MTP1 or IREG1) into circulating blood, where Fe(II) is converted to Fe(III) by a membrane-bound ferroxidase, hephaestin (HEPH), before binding to transferrin and circulating in blood to tissues and cells where iron is needed (Abboud and Haile, 2000; Donovan et al., 2000; McKie et al., 2000); enterocytes may use part of the iron absorbed for their own metabolic needs, whereas excess iron can be sequestered in ferritin for detoxification and storage, and sloughing of these cells at the end of their 3–4 days life span results in excretion of excess duodenal iron (Potten, 1998).

Because of the essential role of duodenal iron absorption in systemic iron homeostasis, duodenal uptake and transfer of iron is regulated by multiple mechanisms. HIF2α has been shown to transcriptionally regulate the expression of iron transporters in iron deficiency and anemia conditions (Majmundar et al., 2010; Shah and Xie, 2013). There are hypoxia responsive elements (HREs) in the promoters of *FPN1*, *DMT1*, and *DCYTB* (also known as *CYBRD1*) and the physiological importance of these HRE–HIF interactions has been demonstrated in the duodenum of *Hif2α* conditional knockout mice, where expression of FPN1, DMT1, and DCYTB does not increase in response to iron deficiency, suggesting that HIF2α has an important physiological role in transcriptional regulation of iron homeostasis (Mastrogiannaki et al., 2009; Shah et al., 2009). Secondly, hepcidin regulates the expression of FPN1 on the basolateral membrane of enterocytes and thereby adjusts iron export from enterocytes into blood (Ganz and Nemeth, 2011, 2012a). Hepcidin is a systemic iron regulatory hormone that is secreted mainly by the liver. Circulating hepcidin can bind FPN1 on the plasma membrane and induce its ubiquitination, internalization and degradation, and thereby reduce iron influx into blood in a feedback manner (Nemeth et al., 2004). Dysregulation of the hepcidin-FPN1 interaction causes the systemic iron overload disease, hemochromatosis, which highlights its significance in systemic iron homeostasis. Thirdly, the intracellular IRP/IRE machinery also regulates duodenal iron absorption, and IRP-related regulation will be discussed later in this review.

Unlike intestinal epithelial cells, other cells *in vivo* use holo-transferrin (transferrin bearing two ferric iron) as the major iron source, and cells absorb iron through the transferrin (Tf)/transferrin receptor (TfR1) cycle. Holo-transferrin in the circulation binds to TfR1 on plasma membranes to form TfR1/Tf/Fe complexes which then internalize to endosomes, whereupon acidification of endosomes induces the release of Fe(III) from Tf. Fe(III) undergoes reduction to Fe(II) by the endosomal ferrireductase STEAP3 before being transported by DMT1 across endosomal membranes into cytosol (Fleming et al., 1998; Ohgami et al., 2005). DMT1 may also directly transport non-transferrin-bound iron (NTBI) into cells *in vivo*, especially in conditions including hemochromatosis and hemolytic anemia when serum iron concentrations exceed the binding ability of transferrin, and therefore NTBI accumulates (Sarkar, 1970; Chua et al., 2004). Once inside the cytosol, part of the iron is taken up by mitochondria and used for heme and iron–sulfur cluster synthesis; excess iron can be exported out of the cells by the iron exporter FPN1, and much extra iron is sequestered in ferritin for detoxification and storage. Ferritin can store up to 4500 iron atoms in a spherical structure formed by 24 subunits of H- and L- ferritin, which self-organize in different ratios, depending on the tissue (Theil, 2012). The IRP/IRE machinery coordinates cellular iron absorption, export, utilization and storage, and thereby regulates intracellular iron homeostasis

## REGULATION OF INTRACELLULAR IRON HOMEOSTASIS BY THE IRP/IRE MACHINERY

The IRP/IRE machinery registers intracellular iron levels, and coordinates iron absorption, export, utilization and storage, providing the fundamental machinery for regulation of intracellular iron metabolism (Rouault, 2006, 2013; Wallander et al., 2006; Muckenthaler et al., 2008). The IRE is a conserved stem-loop structure in the untranslated region (UTR) of target mRNAs. A typical loop has six nucleotides with the sequence of CAGUGN, in which the first C and the fifth G are believed to form a base-pair that stabilizes the structure (Sanchez et al., 2011). The six-nucleotide loop is connected to a stem that is separated into an upper and lower part by an unpaired bulge C residue that divides the stem in the middle. IRE sequences are highly conserved, and mutations of the IRE can cause iron dysregulation and diseases, suggesting a significant role of IRP/IRE regulation in iron homeostasis (Kato et al., 2001; Ismail et al., 2006). IREs are recognized and bound by IRP proteins, but the effect of IRP binding depends on the position of the IRE in the mRNA targets. If an IRE is located in the 5′UTR of target mRNAs, IRP binding can inhibit the translation of such target mRNAs, including L- and H-ferritin (iron storage protein) (Theil, 1990), FPN1 (iron export protein; Abboud and Haile, 2000; Donovan et al., 2000; McKie et al., 2000), erythroid 5-aminolevulinate synthase (eALAS or ALAS2, the first enzyme for heme synthesis; Dandekar et al., 1991), mitochondrial aconitase (ACO2, energy production; Kim et al., 1996; Schalinske et al., 1998), HIF2α (erythropoiesis and hypoxia response; Sanchez et al., 2007), and *Drosophila* succinate dehydrogenase (SDH, citric acid cycle and mitochondrial electron transport chain; Kohler et al., 1995; Melefors, 1996). Conversely, if IREs are present in the 3′UTR of target mRNAs, IRP binding can increase their expression by stabilizing the mRNAs that include TfR1 and DMT1 (iron import proteins; Garrick et al., 2003). In summary, when activated by iron deficiency, IRPs bind the IREs of target mRNAs to increase iron absorption and decrease iron export, iron utilization and iron storage, thereby maintaining appropriate intracellular iron concentrations.

## IRON REGULATORY PROTEINS

Iron regulatory proteins are soluble cytosolic proteins that alter their activities according to intracellular iron levels. There are two IRP proteins in mammalian cells, IRP1 and IRP2, which share 56% sequence identity. In addition, IRP2 has a cysteine-rich 73 amino acid insertion in its N-terminal, but the function of the insertion is not clear yet (Pantopoulos, 2004). Both IRP1 and IRP2 are ubiquitously expressed, with IRP1 highly expressed in the kidneys, liver and brown fat, and IRP2 highly expressed in the central nervous system (Meyron-Holtz et al., 2004). IRP1 and IRP2 are regulated by different mechanisms. IRP1 is a bifunctional enzyme. In iron-replete conditions, IRP1 acquires a [4Fe–4S] cluster in its active cleft and displays cytosolic aconitase activity that catalyzes the conversion of citrate and isocitrate in the cytosol, which probably enhances NADPH generation and lipid synthesis (Tong and Rouault, 2007). In iron-deficient conditions, IRP1 loses its iron–sulfur cluster and acquires IRE-binding activity. The iron–sulfur cluster functions as the iron sensor of IRP1 that endows the protein with the ability to register intracellular iron concentration and adjust its activity accordingly (Rouault, 2006; Medenbach et al., 2011). In contrast to the bifunctional enzyme activity of IRP1, IRP2 does not have an iron–sulfur cluster and lacks aconitase activity, and its activity is regulated by ubiquitination and proteasomal degradation (Rouault, 2009; Salahudeen et al., 2009; Vashisht et al., 2009). Evidence from two independent groups demonstrated that IRP2 is targeted for proteasomal degradation by an E3 ubiquitin ligase complex that contains an F-box protein, FBXL5 (Salahudeen et al., 2009; Vashisht et al., 2009). FBXL5 has a hemerythrin domain that likely binds iron and oxygen, enabling it to function as a regulatory switch that determines the stability of FBXL5, and consequently regulates E3 ubiquitin ligase activity (Chollangi et al., 2012). In brief, iron deficiency and hypoxia destabilize FBXL5 protein, decrease the activity of the E3 ubiquitin ligase, and thereby increase IRP2 activity. *Fbxl*5^-/-^ mice were embryonic lethal, and the lethality could be rescued by deletion of *Irp2*, suggesting that the lethality is likely caused by augmented expression of IRP2 protein, a result that underscores the essential role of FBXL5-E3 ubiquitin ligase in regulation of IRP2 expression (Moroishi et al., 2011). IRP1 is also a target of the FBXL5-E3 ubiquitin ligase complex, and after it loses its [4Fe-4S] cluster by mutation of three cystine residues, IRP1 is down-regulated likely by the FBXL5-mediated proteasomal degradation pathway (Salahudeen et al., 2009; Vashisht et al., 2009). Because IRP1 is relatively stable in the cytosolic aconitase form at iron-replete conditions when FBXL5-E3 ubiquitin ligase is active, the physiological significance of the FBXL5-mediated proteasomal degradation pathway on IRP1 expression is still elusive (Recalcati et al., 2006).

## PHYSIOLOGICAL SIGNIFICANCE OF IRP1 AND IRP2

The IRP/IRE machinery maintains intracellular iron homeostasis and plays a crucial role in development and normal physiology. Animals bred to lack both alleles of *Irp1* and *Irp2* are not viable, and further analyses have shown that embryos at the blastocyst stage display brown color and abnormal morphologies, supporting an essential role of IRPs in early development, before implantation of the embryo (Smith et al., 2006). In contrast, mice with either Irp1 or Irp2 deficiency are viable and fertile, suggesting that Irp proteins can compensate for the loss of one another and are functionally redundant (Meyron-Holtz et al., 2004). The essential role of Irp proteins is also highlighted by conditional knockout experiments which have shown that lack of Irp1 and Irp2 in the intestine of mice results in early death at around 4 weeks of age likely due to intestinal malabsorption and dehydration, and lack of Irp1 and Irp2 in hepatocytes causes liver failure and death of animals within 12 days postpartum (Galy et al., 2008, 2010). Conditional deletion of both *Irp1* and *Irp2* in hepatocytes compromises iron–sulfur cluster and heme synthesis, and impairs mitochondrial functions, suggesting an essential role of Irps in supplying iron to mitochondria to maintain respiration. Though adult mice with ligand-induced deletion of both *Irp1* and *Irp2* in duodenal enterocytes responded well to iron loading and erythropoietic stimulation, these mice displayed reduced iron absorption and iron accumulation in duodenal enterocytes, suggesting that Irps play an important role in adjusting duodenal iron absorption by regulating ferritin expression to create a ferritin dependent “mucosal block” (Galy et al., 2013).

## PHYSIOLOGICAL SIGNIFICANCE OF IRP2

**FIGURE 1:**
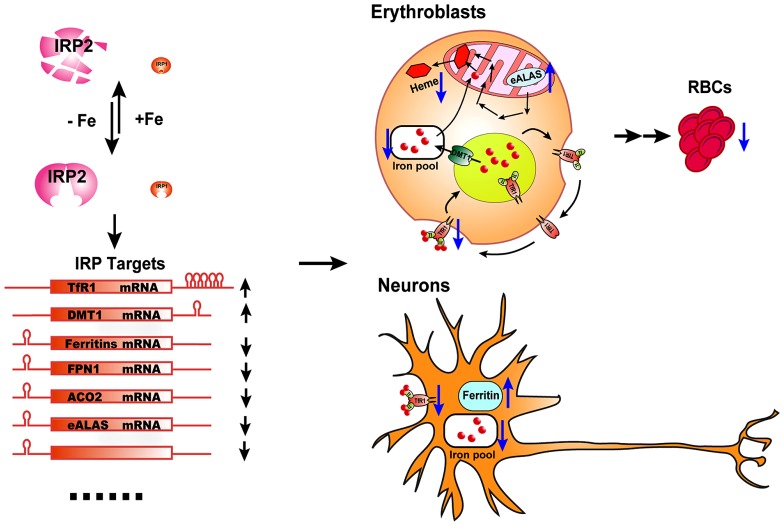
**The physiological effect of IRP2 deficiency *in vivo.*** IRP2 is the predominant IRP protein in erythroblasts and neurons. According to intracellular iron status, IRP2 protein levels are regulated by the FBXL5-dependent proteasomal degradation pathway. By binding to the IREs in target transcripts of iron metabolism genes, IRP2 increases the expression of iron importers TfR1 and DMT1, and decreases the expression of the iron storage protein, ferritin, the iron exporter FPN1, and some iron utilization-related genes including ACO2 and eALAS, as well as other potential target genes, to maintain optimal intracellular iron levels. In erythroblasts, IRP2 deficiency decreases the expression of TfR1, resulting in insufficient import of iron by the Tf/TfR1 cycle to support heme biosynthesis, and derepresses eALAS expression, leading to protoporphyrin IX accumulation and microcytic hypochromic anemia. In neurons, IRP2 deficiency decreases the expression of the iron importer TfR1 and increases the expression of the ferritin. The accumulation of iron by ferritin depletes biologically available iron from the cytosol and leads to functional iron deficiency, mitochondrial dysfunction and neuronal degradation.

Though deletion of both *Irp1* and *Irp2* is embryonic lethal, mice with deletion of either *Irp1* or *Irp2* are viable and fertile (Meyron-Holtz et al., 2004). Irp2 deficiency causes iron misregulation in the duodenum, central nervous system, and most prominently in motor neurons of spinal cord; misregulation is characterized by increased expression of ferritin, decreased expression of TfR1, and significant iron accumulation in the duodenal mucosa and neurons throughout the brain (LaVaute et al., 2001). Iron misregulation correlates with axonal degeneration and neuronal death in the brain and spinal cord, and animals in late stages of adulthood display a movement disorder characterized by abnormal gait, tremor, and hind-limb paralysis (LaVaute et al., 2001; Jeong et al., 2011). Compared with *Irp2*^-/-^ mice, loss of one more copy of *Irp1* in *Irp1*^+^^/^^-^Irp2^-/-^ mice exacerbates the neurodegenerative symptoms as evidenced by the increased myelin dense bodies in the ventral spinal cord in the region where motor neurons are found (a hallmark of neurodegeneration), increased stress markers, increased macrophage infiltration, and decreased diameters of motor neuronal axon bundles, suggesting that there is a dosage effect of Irp1 and Irp2 deficiency and confirming that neurodegenerative symptoms of *Irp2*^-/-^ mice are caused by iron dysregulation. Deficiency of Irp*2* increases the expression of the iron storage protein ferritin and decreases expression of the iron importer TfR1, leading to functional iron deficiency (lack of biologically available iron) in conjunction with apparent ferric iron overload caused by sequestration of iron in ferritin; and the notion that there is functional iron deficiency is also supported by deficiency of mitochondrial complex I/II activity (**Figure 1**; Jeong et al., 2011). The neurodegenerative symptoms of *Irp2*^-/-^ mice were improved by activation of IRP1 activity with oral treatment by Tempol (4-hydroxy-2,2,6,6-tetramethylpiperidin-1-oxyl), a scavenger of reactive oxygen species, which was shown to destabilize the iron–sulfur cluster ligand of IRP1 and restore its IRE-binding activity (Ghosh et al., 2008; Wilcox and Pearlman, 2008). The neurodegenerative symptoms of *Irp2*^-/^^-^ mice were also improved by deletion of one allele of ferritin-H chain, which limited iron sequestration and increased intracellular iron availability. These studies support that lack of IRE-binding activity of IRP2 and subsequent derepression of ferritin translation cause the neurodegenerative diseases of Irps deficient animals (Ghosh et al., 2008; Jeong et al., 2011). The neurodegenerative symptoms of *Irp2*^-/^^-^ mice were also assessed by other two groups in independent *Irp2*^-/^^-^ mouse colonies. Compared with the neurodegenerative symptoms characterized by ataxia, tremor and motor impairment associated with iron deposit in the white matter and nuclei throughout the brain in the *Irp2*^-/^^-^ mice of our group (LaVaute et al., 2001; Jeong et al., 2011), one group found that while their *Irp2*^-/^^-^ mice displayed discrete impairment of balance and/or motor coordination, they did not find iron deposit with Perl’s-DAB iron staining and did not find evidence of neuronal degeneration in the brain (Galy et al., 2006); the other group found that their *Irp2*^-/^^-^ mice had significant locomotor dysfunction and increased iron deposits in the cortex, mid-brain and cerebellum of *Irp2*^-/^^-^ mice, but they did not find cellular degeneration with Fluoro-Jade staining (Zumbrennen et al., 2009). The locomotor dysfunction displayed by these three *Irp2*^-/^^-^ mouse colonies confirms the significant role of Irp2 in mouse neuronal system and also suggests that the discrepancy is likely from either different mouse genetic backgrounds or differences in experimental assessement, but not due to off-target effects as was suggested before (Galy et al., 2006). Since IRP1 and IRP2 have redundant functions, deletion of one copy of *Irp1* in *Irp2*^-/^^-^ mice probably could exacerbate the neurodegenerative symptoms and provide more information of IRPs function in the other two *Irp2*^-/^^-^ mouse colonies. Further, conditional knockout of both *Irp1* and *Irp2* in neurons as in hepatocytes and enterocytes could shed light on the role of IRPs in neuronal system, although complete loss of Irps has been lethal in several settings (Smith et al., 2006; Galy et al., 2008, 2010, 2013).

*Irp2*^-/-^ mice also display microcytic hypochromic anemia (Cooperman et al., 2005; Galy et al., 2005). Erythroblasts of *Irp2*^-/-^ mice have decreased TfR1 expression and iron levels, and increased protoporphyrin IX levels, supporting an iron-limited erythropoiesis model. Irp2 deficiency destabilizes TfR1 mRNA, and lack of TfR1 protein causes iron deficiency and eventually leads to microcytic anemia. The microcytic anemia of *Irp2*^-/-^ mice suggests that Irp2 plays a dominant role in regulating iron homeostasis in erythroid cells (**Figure 1**). While the neurodegenerative symptoms of *Irp2*^-/-^ are corrected by Tempol treatment, the treatment did not improve the microcytic anemia (Ghosh et al., 2008). Analogously, while Tempol treatment significantly converted Irp1 fraction from cytosolic aconitase form to IRE-binding form in forebrain lysate, which likely compensated the loss of IRE-binding activity in the brain of *Irp2*^-/-^ mice, Tempol treatment did not increase the IRE-binding activity of Irp1 in erythroblasts, likely because Irp1 is already mainly in the IRE-binding form in erythroblasts (Ghosh et al., 2008).

In addition to the microcytic anemia and neurodegenerative symptoms, *Irp2*^-/-^ mice displayed iron overload in duodenum and liver, but interestingly, these animals also displayed iron deficiency in the spleen and bone marrow (Cooperman et al., 2005; Galy et al., 2005). Conditional deletion of *Irp2* in mouse duodenal enterocytes and liver hepatocytes repeats the iron overload phenotype of duodenum and liver, suggesting that iron overload in these two tissues is likely due to cell-autonomous functions of Irp2 deficiency in enterocytes and hepatocytes (Ferring-Appel et al., 2009). In contrast, conditional deletion of *Irp2* in mouse splenic macrophages did not produce the splenic iron deficiency seen in *Irp2*^-/-^ mice, suggesting that the splenic iron deficiency of *Irp2*^-/-^ mice is likely secondary to iron misregulation in other cell types. Considering that (i) both spleen and bone marrow macrophages of *Irp2*^-/-^ mice display iron deficiency, (ii) both spleen and bone marrow are erythropoietic tissues, (iii) splenic macrophages play an essential role in recycling iron from senescent RBC, the iron deficiency of splenic macrophages probably results from reduced acquisition of iron from red cell turnover in anemic *Irp2*^-/-^ mice. Conditional deletion of *Irp2* in erythroblasts might shed light on the pathogenesis of splenic iron deficiency.

## PHYSIOLOGICAL SIGNIFICANCE OF IRP1

Though both IRP1 and IRP2 are ubiquitously expressed and show similar binding affinities for target IREs in *in vitro* experiments, *Irp1*^-/-^ and *Irp2*^-/-^ mice display different phenotypes (Henderson et al., 1993; Kim et al., 1995). Because the protein levels and IRE binding activities of IRP1 in electrophoresis mobility shift assays are much higher than those of IRP2 in cultured cells and in certain tissues such as liver and kidneys, the physiological significance of IRP1 has been relatively elusive. In contrast to the neurodegeneration and anemia symptoms of *Irp2*^-/-^ mice, *Irp1*^-/-^ mice initially appeared asymptomatic and the physiological significance of IRP1 in iron metabolism was unclear. However, evidence from *Irp1*^-/-^ mice has recently emerged, which indicates that IRP1 plays an essential role in regulation of systemic iron homeostasis and erythropoiesis.

### PHYSIOLOGICAL SIGNIFICANCE OF IRP1 IN ERYTHROPOIESIS AND SYSTEMIC IRON HOMEOSTASIS

In contrast to *Irp2*^-/-^ mice that develop microcytic hypochromic anemia with hematocrits of about 36%, adult *Irp1*^-/-^ mice produce more RBCs than wild type animals (hematocrit ~50 vs. ~45%, *Irp1*^-/-^ vs. wild type; Cooperman et al., 2005; Ghosh et al., 2013). High hematocrits were also observed in 4–6 weeks old mice of a different colony where two research groups found that *Irp1*^-/-^ mice had hematocrits of more than 70% (Pawlus and Hu, 2013; Wilkinson and Pantopoulos, 2013). However, these researchers found that hematocrits of *Irp1*^-/-^ mice decreased with age, and the difference of hematocrits between *Irp1*^-/-^ and wild type mice disappeared after 8 weeks of age. We also checked the hematocrits of *Irp1*^-/-^ mice in different ages (from 4 weeks to 14 months old), but did not find a significant decrease of the hematocrits of mice older than 8 weeks of age (Zhang et al., unpublished data), and the reason for the inconsistent observations is not yet clear. More striking results are obtained from *Irp1*^-/-^ mice that are maintained on an iron-deficient diet (Ghosh et al., 2013). In contrast to the general observation that iron deficiency causes iron deficiency anemia, maintenance on an iron-deficient diet significantly increased the hematocrits of *Irp1*^-/-^ mice from about 50% to more than 60%. Serum erythropoietin (EPO) levels of *Irp1*^-/-^ mice were more than seven-fold higher than that in wild type mice maintained on an iron-deficient diet, and animals with these high EPO levels developed splenomegaly and increased splenic erythropoiesis, supporting a model that there is EPO-dependent extracellular erythropoiesis in *Irp1*^-/-^ mice. HIF2α is the master transcription factor *in vivo* that regulates EPO levels and subsequent RBC production according to both hypoxia and iron status (Semenza, 2009; Haase, 2013). HIF2α mRNA has an IRE in its 5′UTR with an initially unknown significance (Sanchez et al., 2007). In the renal carcinoma cell line, 786-O cells, HIF2α expression is increased by *Irp1* knockdown but not *Irp2* knockdown, suggesting that Irp1 probably plays a major function in repressing HIF2α translation by binding to its 5′IRE in these particular cells (Zimmer et al., 2008). Irp1 but not Irp2 deficiency significantly increased the percentage of HIF2α mRNA found in the polysomal fractions of mouse kidneys and liver, proving that Irp1 but not Irp2 represses HIF2α mRNA translation in cells of these two tissues *in vivo* (Anderson et al., 2013; Wilkinson and Pantopoulos, 2013). Thus, in *Irp1*^-/-^ mice, Irp1 deficiency derepresses HIF2α translation, which transcriptionally increases EPO expression and subsequently drives red blood production, leading to polycythemia as a consequence (Anderson et al., 2013; Ghosh et al., 2013; Wilkinson and Pantopoulos, 2013). Notably, *Irp1*^-/-^ mice have severe iron deficiency as evidenced by low serum iron levels and reduced stainable tissue iron compared with wild type animals, which is likely caused by increased erythropoiesis that channels iron into the production of red cells, and consequently depletes Tf bound iron in blood and tissue iron stores.

**FIGURE 2:**
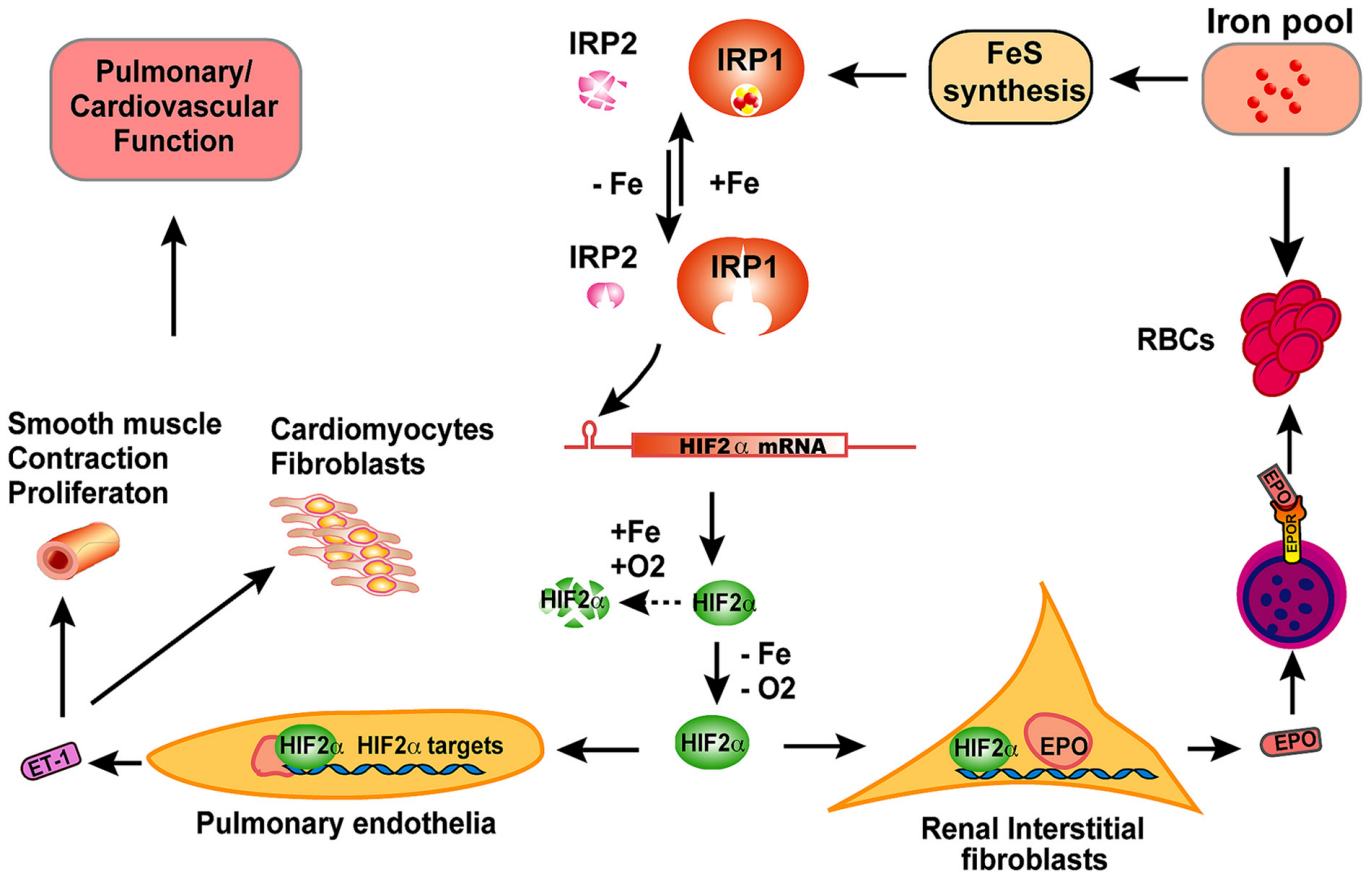
**The scheme of physiological significance of IRP1 *in vivo.*** IRP1 is the predominant IRP protein in renal interstitial fibroblasts and pulmonary endothelial cells. In iron-replete conditions, IRP1 ligates an iron–sulfur cluster and displays cytosolic aconitase activity; in iron-depleted conditions, IRP1 loses its iron–sulfur cluster and binds to IREs of target mRNAs to regulate expression of iron metabolism related genes. By binding to the 5′IRE of HIF2α mRNA, IRP1 regulates HIF2α expression according to iron and oxygen status, and thereby fine-tunes the levels of HIF2α protein in cooperation with prolyl hydroxylases and the Von Hippel–Lindau mediated proteasomal degradation pathway. In hypoxia or iron deficiency conditions, HIF2α protein is stabilized due to inactivation of prolyl hydroxylases, and then translocates to nucleus and transcriptionally increase erythropoietin (EPO) expression. Circulating through blood, EPO binds to EPO receptors on erythroblasts and stimulates erythroblasts to produce red blood cells (RBC), a process that consumes large amount of iron. When too much iron is consumed and systemic iron levels are low, IRP1 will be activated to reduce HIF2α expression and restrict RBC production. By this feedback mechanism, IRP1 regulates the balance between systemic iron homeostasis and erythropoiesis. In pulmonary endothelial cells, IRP1 regulates HIF2α translation and subsequently regulates the expression of endothelin-1 (ET-1), a peptide hormone that regulates pulmonary vascular contraction and the proliferation of smooth muscle cells, cardiomyocytes, and fibroblasts. Though the exact mechanism remains to be elucidated, IRP1 deficiency causes pulmonary hypertension and cardiovascular diseases in mice likely by derepression of HIF2α expression, increase of ET-1 levels and most probably other HIF2α targets also.

The polycythemia of *Irp1*^-/-^ mice and its exacerbation by iron deficiency suggest a crucial role of Irp1 in regulating systemic iron levels and erythropoiesis (**Figure 2**). HIF2α is regulated at the post-translational level by prolyl hydroxylases (PHDs) that use oxygen and iron as substrates to hydroxylate HIF2α at two conserved proline residues. Following hydroxylation, HIF2α undergoes ubiquitination by the von Hippel–Lindau E3 ubiquitin ligase and is subsequently degraded through the proteasomal pathway (Majmundar et al., 2010; Lee and Percy, 2011; Yoon et al., 2011; Haase, 2013). Hence, HIF2α can sense hypoxia and iron deficiency, and then increases EPO expression and drives red blood production, a process that consumes large amounts of iron. The regulation of HIF2α translation by Irp1 provides a safeguard that prevents erythropoiesis from consuming too much iron to deplete systemic iron. While HIF2α senses hypoxia and stimulates EPO expression and RBC production, Irp1 fine-tunes HIF2α expression to ensure that there is enough iron available for iron–sulfur cluster synthesis; if intracellular iron levels are low, iron–sulfur cluster synthesis is impaired and IRP1 will be converted to the IRE-binding form, which represses HIF2α translation, and thereby decreases RBC production to restore systemic iron balance (**Figure 2**). *Irp1*^-/-^ mice on an iron-deficient diet have increased polycythemia, severe iron deficiency, and sudden death due to peritoneal hemorrhage, which emphasizes the crucial role of Irp1 in systemic iron homeostasis and erythropoiesis (Ghosh et al., 2013). Maintenance of *Irp1*^-/-^ animals in 10% oxygen for 3 weeks can increase the hematocrits of *Irp1*^-/-^ mice to as high as 80%, compared to 65% in wild type mice, highlighting the significance of IRP1 in systemic iron homeostasis and erythropoiesis under hypoxia (Zhang et al., unpublished data). Mice that inducibly express a constitutively active IRP1 mutant (IRP1^*^) develop macrocytic anemia, probably due to impaired erythropoiesis as displayed by increased erythroid progenitor and decreased numbers of mature cells (Casarrubea et al., 2013). Erythroblasts of IRP1^*^ mice have higher TfR1 expression compared to wild type animals, which could potentially cause iron overload and impair normal erythropoiesis. In addition, high levels of Irp1 are expected to repress HIF2α translation and subsequently reduce EPO levels and RBC production. The macrocytic anemia of IRP1^*^ highlights the importance of IRP/IRE balance in iron and erythropoiesis homeostasis.

In addition to its function in the regulation of erythropoiesis and systemic iron homeostasis, IRP1 probably also plays a role in coordinating intracellular Fe-S cluster and heme synthesis in erythroid cells (Wingert et al., 2005; Ye and Rouault, 2010; Chung et al., 2014). Because IRP1 has a [4Fe-4S] cluster as the sensor to adjust its IRE-binding activity, IRP1 can sense Fe-S cluster deficiency and potentially repress the translation of ALAS2 (eALAS) by binding to a IRE in its 5′UTR. As ALAS2 is the first enzyme of the heme synthesis pathway, translational repression by IRP proteins can coordinate the synthesis of Fe-S clusters and heme (Dandekar et al., 1991; Wingert et al., 2005; Chung et al., 2014). Deficiency of glutaredoxin 5 (GLRX5), a scaffold protein required for mitochondrial Fe-S cluster synthesis, activated IRP1, which inhibited ALAS2 translation and subsequently led to anemia in zebrafish, suggesting a role of IRP1 in coordinating Fe-S cluster and heme synthesis (Wingert et al., 2005). Consistent with the zebrafish studies, RNAi knockdown of GLRX5 in K562 cells markedly reduced ALAS2 expression, and deficiency of GLRX5 in a patient caused by an intronic mutation that caused missplicing significantly increased the IRE-binding activity of IRP1, and caused sideroblastic anemia (Camaschella et al., 2007; Ye et al., 2010). IRP1 deficiency significantly increased protoporphyrin levels in erythroid cells with heterozygous deficiency of mitoferrin1, the major mitochondrial iron importer in erythroid cells, also suggesting that IRP1 is an important link between Fe-S cluster and heme synthesis (Shaw et al., 2006; Chung et al., 2014). The evidence suggests that IRP1 likely plays an important role in orchestrating the heme and Fe-S cluster synthesis in erythroid precursors; however, the role of IRP2 was not really tested, considering that: (1) Zebrafish do not have an IRP2 homolog (Wingert et al., 2005); (2) The effect of IRP2 deficiency on protoporphyrin levels in erythroid precursors with heterozygous mitoferrin1 deficiency was not checked (Chung et al., 2014); (3) IRP2 expression was also significantly increased in GLRX5 deficient fibroblasts (Ye et al., 2010); (4) *Irp2*^-/-^ mice, but not *Irp1*^-/-^ mice, have increased protoporphyrin IX levels (Cooperman et al., 2005). Thus, the roles of IRP1 and IRP2 in coordinating heme and Fe-S cluster synthesis in erythroid precursors may require further investigation.

### PHYSIOLOGICAL SIGNIFICANCE OF IRP1 IN PULMONARY AND CARDIOVASCULAR SYSTEM

In addition to the essential role of IRP1 in maintaining erythropoiesis and systemic iron homeostasis, IRP1 also plays an important role in pulmonary and cardiovascular system (**Figure 2**). *Irp1*^-/-^ mice displayed cardiac hypertrophy and pulmonary hypertension, two severe human diseases with unclear pathogenesis (Ghosh et al., 2013). HIF2α has been previously implicated in the pathogenesis of pulmonary hypertension. Heterozygous deficiency of HIF2α protects mice against developing pulmonary hypertension and right ventricular dysfunction during prolonged hypoxia, suggesting that HIF2α is involved in pathogenesis of pulmonary hypertension (Brusselmans et al., 2003). Chuvash polycythemia is a hereditary disease caused by VHL^R200W^ mutation that disrupts the degradation pathway of HIFα and consequently increases HIF2α protein to high levels (Hickey et al., 2007). Chuvash polycythemia patients and a corresponding mouse model with a VHL^R200W^ mutation develop polycythemia and pulmonary hypertension, and the pulmonary hypertension of the mouse model is improved by deletion of one allele of *Hif2α*, suggesting that there is a pathogenic role of HIF2α in this disease (Bushuev et al., 2006; Hickey et al., 2010; Formenti et al., 2011). Endothelin-1, a potent vasoconstrictor and HIF target, is significantly increased in Chuvash mice and could be a downstream effector molecule in the pathogenesis of pulmonary hypertension (Bushuev et al., 2006; Hickey et al., 2010; Thorin and Clozel, 2010; Shao et al., 2011). HIF2α expression is significantly increased in cultured primary endothelial cells of *Irp1*^-/-^ mice, and endothelin-1 expression is also significantly increased in lung tissues of *Irp1*^-/-^ mice, supporting a pathogenic role of these molecules in the pulmonary hypertension (Ghosh et al., 2013). However, preliminary results of *Irp1*^-/-^ mice did not reveal significant pulmonary vascular remodeling, in contrast to the Chuvash and the prolonged-hypoxia mouse models, and thus the mechanism underlying the pulmonary hypertension of *Irp1*^-/-^ mice is not very clear yet. Low iron treatment significantly increases EPO expression and exacerbates the polycythemia of *Irp1*^-/-^ mice, which could be attributed to the stabilization of HIF2α by iron deficiency; in contrast, the iron-deficient diet did not exacerbate the pulmonary hypertension of *Irp1*^-/-^ mice, and the endothelin-1 levels were not altered either, suggesting that there are mechanistic differences in the pathophysiology of polycythemia and pulmonary hypertension. Analysis of the underlying molecular pathophysiology could shed light on the pathogenesis of these diseases. The IRP1/HIF2α interaction in kidneys represents a mechanism that protects systemic iron homeostasis during erythropoiesis by preventing red cells production from depleting systemic iron, whereas the evolutionary rationale for the IRP1/HIF2α interaction in pulmonary vascular function is not yet clear (**Figure 2**).

Maintenance on an iron-deficient diet also significantly decreases life span of *Irp1*^-/-^ mice. From the age of 3 months old, *Irp1*^-/-^ mice on the iron-deficient diet are prone to sudden death in which apparently healthy mice die without warning (Ghosh et al., 2013). Pathological analyses of mouse carcasses revealed that these mice died of abdominal hemorrhage around the perinephric area, suggesting that there is an essential role of IRP1 in the vascular system. Considering that the iron-deficient diet exacerbates the polycythemia of *Irp1*^-/-^ mice by stimulating HIF2α expression, HIF2α probably also plays a role in the peritoneal hemorrhage. Notably, Chuvash polycythemia patients, who have high HIF2α expression, also have increased incidence of thrombotic events, major bleeding episodes, and premature mortality, suggesting that there are probably similar mechanisms operating in Chuvash polycythemia patients and the *Irp1*^-/-^ mouse models (Gordeuk and Prchal, 2006). Coagulation factor VIII (FVIII) is an essential blood-clotting protein, and high levels of FVIII are reported to associate with increased risk of deep vein thrombosis and pulmonary embolism (Jenkins et al., 2012). An alternative transcript variant 2 of FVIII has a potential 5′IRE that, if functional, could derepress FVIII expression in IRP1 deficient animals or patients (Livesey et al., 2012). The expression and role of FVIII in the phenotypes associated with the *Irp1*^-/-^ mouse model warrant investigation.

## DIFFERENT PHENOTYPES OF *Irp*1^-/-^ AND *Irp*2^-/-^ MICE ARE LIKELY CAUSED BY CELL-SPECIFIC EXPRESSION

The relative functions of IRP1 and IRP2 have been a subject of debate since they were identified two decades ago. On one hand, IRP1 and IRP2 are highly conserved in sequence and displayed similar binding affinity to their target in *in vitro* experiments (Henderson et al., 1993; Kim et al., 1995); on the other hand, IRP1 and IRP2 have different regulatory mechanisms with IRP1 functioning as a bifunctional protein and IRP2 being degraded through the proteasomal pathway. The polycythemia and pulmonary hypertension phenotype of *Irp1*^-/-^ mice and the anemia and neurodegeneration phenotype of *Irp2*^-/-^ mice likely support a unique functions of IRP1 and IRP2 in erythropoiesis/cardiovascular regulation and erythroblasts/nervous system, respectively. However, a recent study, which analyzed the endogenous transcripts bound *in vitro* by overexpressed IRP1 and IRP2 proteins with microarray, showed that IRP1 and IRP2 shared 44 transcripts including the transcripts that had been confirmed in literature, i.e., FTL, FTH, TfR1, DMT1, FPN1, ACO2, eALAS, and HIF2α (Sanchez et al., 2011). Derepression of the shared target HIF2α most likely causes the polycythemia and pulmonary hypertension of *Irp1*^-/-^ mice (Anderson et al., 2013; Ghosh et al., 2013; Wilkinson and Pantopoulos, 2013), and dysregulation of the shared targets ferritin, TfR1, and eALAS likely lead to the diseases of *Irp2*^-/-^ mice (LaVaute et al., 2001; Cooperman et al., 2005; Galy et al., 2005; Ferring-Appel et al., 2009; Jeong et al., 2011; Ghosh et al., 2013). IRP1 expression is very high relative to IRP2 in the kidneys and lung, whereas IRP2 expression is very high in the brain, which is consistent with the symptoms of *Irp1*^-/-^ and *Irp2*^-/-^ mice in affected tissues, suggesting that the differences in the phenotypes of *Irp1*^-/-^ and *Irp2*^-/-^ mice are most likely caused by differences in the cell-specific expression of these two proteins (**Figures 1 and 2**; Meyron-Holtz et al., 2004; Ghosh et al., 2008, 2013). Nevertheless, in addition to the 44 targets shared by both IRP1 and IRP2, microarray analysis of IRP-binding transcripts also identified 101 potential IRP1-specific targets and 113 potential IRP2-specific targets. The contribution of these targets in the pathogenesis of *Irp1*^-/-^ and *Irp2*^-/-^ mice is not clear yet, and characterization of these IRP1- or IRP2- specific targets could shed new light on the unique functions of IRP1 and IRP2 in iron metabolism and development. In addition to the different expression profiles of IRP1 and IRP2 in cells and tissues affected by IRP1 and IRP2 deficiency, we have to keep in mind that the expression profiles of other genes including IRP targets as well as other iron metabolism related genes are also unique in each of these cell types and tissues. Since IRP targets share the same IRE-binding protein pool, changes in mRNA expression of each of these IRP targets will create a unique gene expression context and thereby inevitably affect the binding of IRPs to other targets, which eventually leads to the diseases of Irp1 and Irp2 deficiency animals. Since there have been very few attempts to understand iron metabolism in gene expression context (Hower et al., 2009), application of systemic biology methodologies probably could elucidate a better picture of the molecular pathophysiology of disease in the *Irp1*^-/-^ and *Irp2*^-/-^ mice.

## CLINICAL IMPLICATIONS

The IRP/IRE system plays an essential role in iron homeostasis, and dysregulation of IRP/IRE system has been reported to cause many diseases. Mutations in the IRE element of human L-ferritin disrupt the regulation of IRPs on L-ferritin translation and result in high ferritin expression and early onset cataracts, causing hereditary hyperferritinemia cataract syndrome (Ismail et al., 2006). A mutation in the IRE of human H-ferritin causes autosomal dominant iron overload (Kato et al., 2001). Disruption of IRE elements in the mouse FPN1 promoter alters erythropoiesis and iron homeostasis, and induces age-dependent loss of photoreceptors of the retina (Mok et al., 2004a, b; Iacovelli et al., 2009). These diseases and phenotypes highlight the essential role of IRP/IRE machinery in iron metabolism and development. The neurodegeneration and anemia of *Irp2*^-/-^ mice and the polycythemia and pulmonary hypertension of *Irp1*^-/-^ mice underline the essential role of IRP/IRE machinery in regulating cellular and systemic iron homeostasis, and also suggest that mutations of IRP1 and IRP2 could underlie some human diseases. Though patients with diseases attributable to IRP2 mutations have not been identified, such patients could be treated by activating IRP1 with stable nitroxide, Tempol, or other nitric oxide sources, which could compensate for the loss of IRP2 and ameliorate disease, as demonstrated by alleviation of symptoms in Tempol-treated *Irp2*^-/-^ mice (Lipinski et al., 2005; Ghosh et al., 2008; Jeong et al., 2011).

Polycythemia is a severe disease that stresses the cardiovascular system and endangers the lives of patients. A routine treatment for polycythemia is phlebotomy to remove excess blood from patient. The essential role of IRP1 in regulating HIF2α translation, as displayed by *Irp1*^-/-^ mice, suggests that activation of IRP1 by Tempol or nitric oxide could repress HIF2α translation and thereby decrease EPO expression and reduce RBC production. Activation of IRP1 could be a therapeutic strategy to treat Chuvash polycythemia patients. Erythropoiesis-stimulating agents have been widely used to treat anemia of chronic diseases, and the significant role of IRP1 in repressing HIF2α and EPO production suggests that inhibiting the interaction between IRP1 and HIF2α with small molecules could increase HIF2α translation and up-regulate endogenous EPO levels (Zimmer et al., 2008). Phenotypes of *Irp1*^-/-^ mice also suggest that IRP1 mutations could cause idiopathic polycythemia and pulmonary hypertension in some patients, and IRP1 should be screened as a candidate disease gene in these patients. *Irp1*^-/-^ mice provide a novel mouse model for pulmonary hypertension, cardiac hypertrophy and aneurysm, and investigations of this model will provide insights into the molecular mechanisms of these diseases. Iron deficiency anemia is one of the most common diseases worldwide. The translational derepression of HIF2α and subsequent increase of EPO levels in *Irp1*^-/-^ mice prove that IRP1 plays a critical role in balancing erythropoiesis and systemic iron homeostasis during iron deficiency; these observations explain why the first manifestation of iron deficiency is usually anemia.

## FUTURE DIRECTIONS

The phenotypes of *Irp1*^-/-^ and *Irp2*^-/-^ mice provide compelling evidence for the essential role of the IRP/IRE system in systemic iron homeostasis and physiology, suggesting that IRP1 or IRP2 mutations could cause human diseases similar to those discovered in animal models. Screening human patients and identifying IRP1 or IRP2 mutations could deepen our understanding of their roles in human iron metabolism. *Irp1*^-/-^ mice on low iron diet died from peritoneal hemorrhage, and the physiological function of IRP1 in the pulmonary and cardiovascular system is not clear yet. Low iron treatment exacerbates the polycythemia but not pulmonary hypertension, and the molecular mechanism remains to be elucidated. Pharmacological activation of IRP1 could be a therapeutic strategy to treat Chuvash polycythemia and pulmonary hypertension, and inhibition of the interaction between IRP1 and HIF2α could increase EPO production and potentially treat anemia, and thus modulations of IRP1 activity should be investigated further. As IRP1 is a bifunctional enzyme, *Irp1*^-/-^ mice lose both the IRE-binding activity and cytosolic aconitase activity, and the contribution and significance of cytosolic aconitase activity of IRP1 *in vivo* merits further investigation. Microarray analysis of IRP1- and IRP2- associated mRNAs identified 35 novel mRNAs that can bind both IRP1 and IRP2, as well as mRNAs bind exclusively to either IRP1 or IRP2, and experimental analysis and characterization of those mRNAs could advance our understanding of the function of IRP/IRE system in systemic iron metabolism (Sanchez et al., 2011).

## Conflict of Interest Statement

The authors declare that the research was conducted in the absence of any commercial or financial relationships that could be construed as a potential conflict of interest.
